# Supplementary material for: Fast evolutionary turnover and overlapping variances of sex-biased gene expression patterns defy a simple binary sex classification of somatic tissues
Source: eLife. 2025 Sep 17;13:RP99602. doi: 10.7554/eLife.99602 (PMC12443475; doi:10.7554/eLife.99602)
Supplement: Figure 1—source data 4. — Based on the values provided in Supplementary Data D1, all plotted in log-scale. [file elife-99602-fig1-data4.pdf]

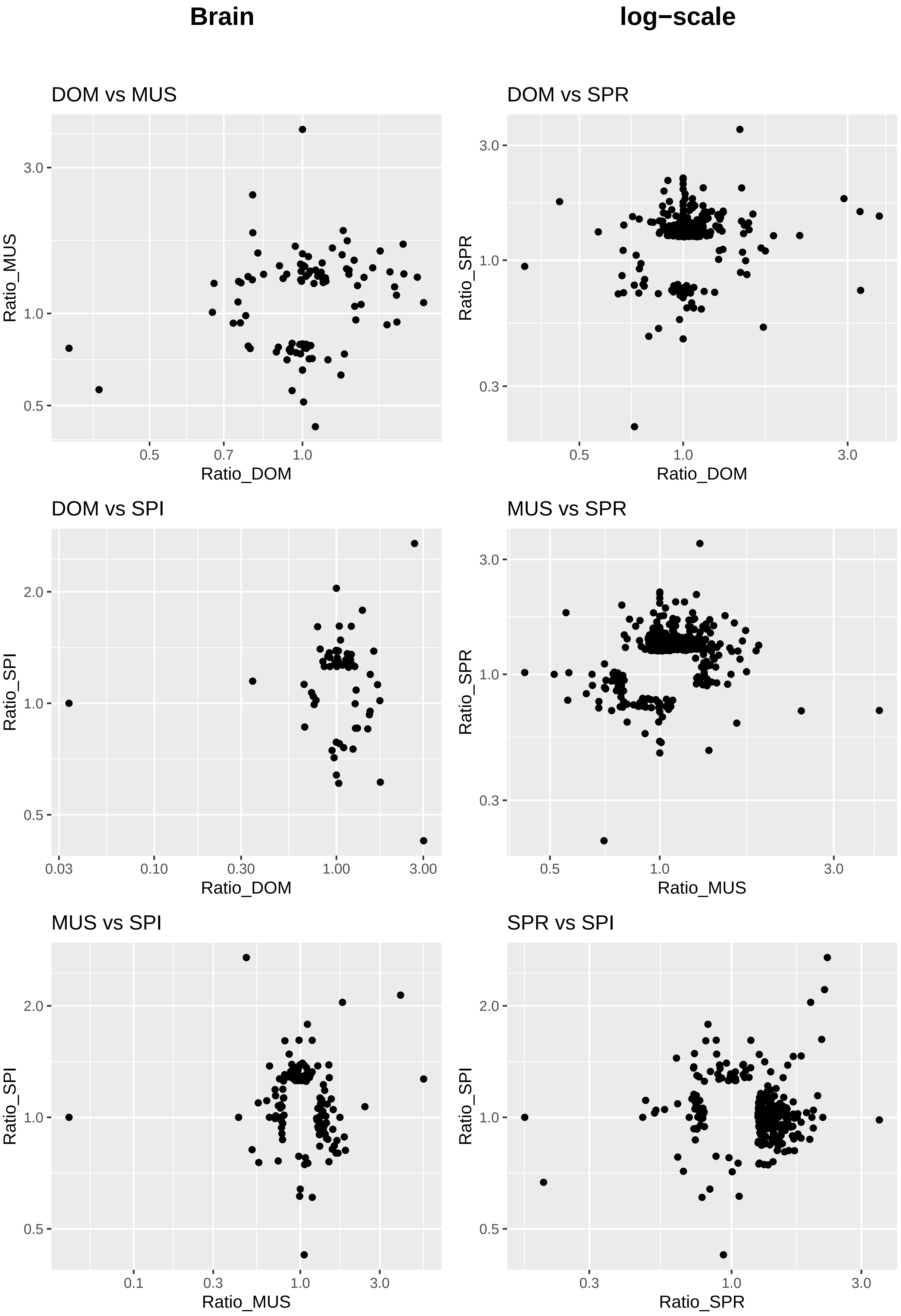
Brain
log−scale
DOM vs MUS
Ratio_MUS
Ratio_DOM
0.5
0.7
1.0
3.0
DOM vs SPR
Ratio_SPR
Ratio_DOM
0.3
1.0
3.0
0.5
DOM vs SPI
Ratio_SPI
Ratio_DOM
0.03
0.10
0.30
1.00
3.00
0.5
1.0
2.0
MUS vs SPR
Ratio_SPR
Ratio_MUS
MUS vs SPI
Ratio_SPI
Ratio_MUS
0.1
0.3
SPR vs SPI
Ratio_SPI
Ratio_SPR

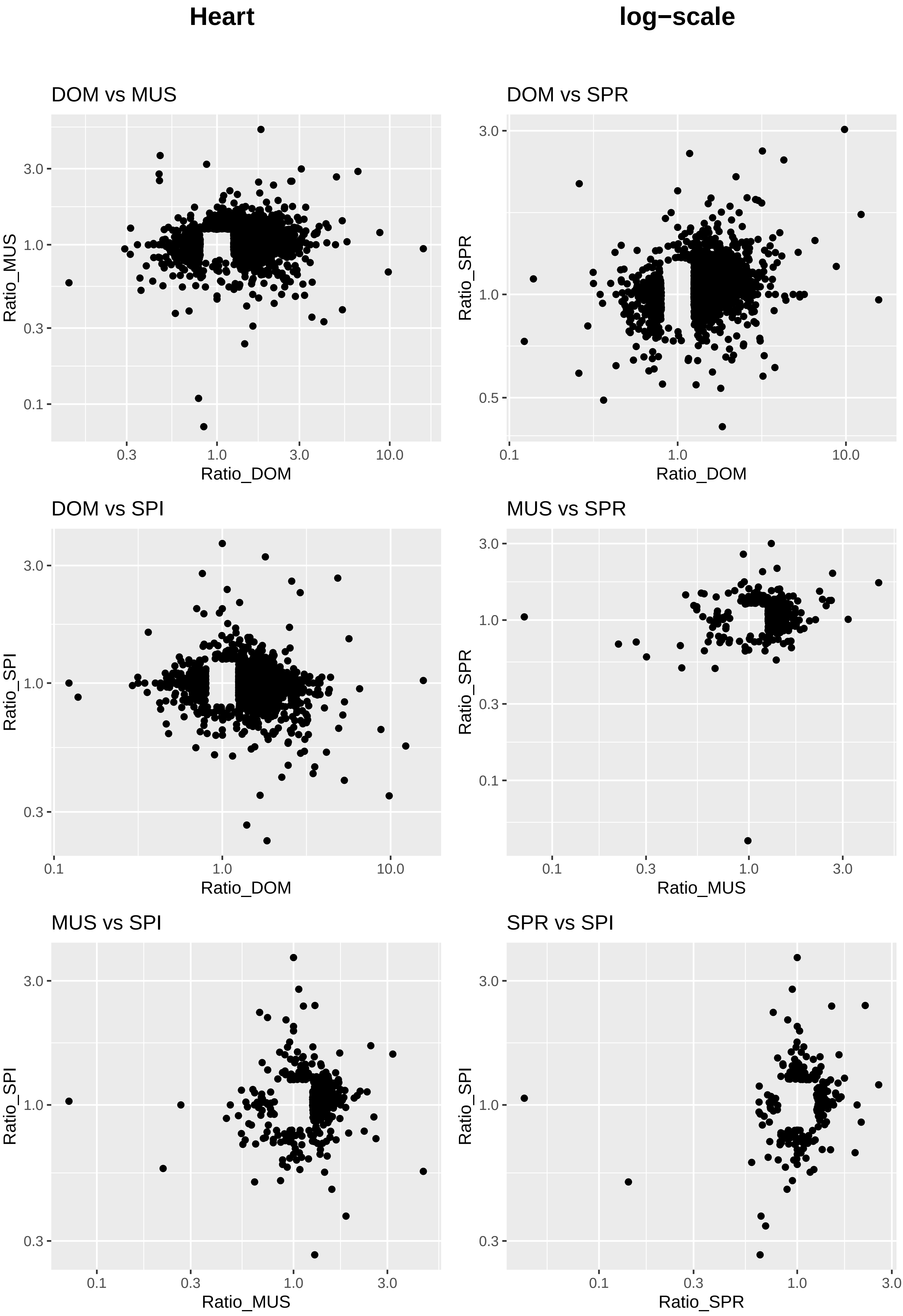
Heart
log−scale
DOM vs MUS
Ratio_MUS
Ratio_DOM
DOM vs SPR
Ratio_SPR
Ratio_DOM
DOM vs SPI
Ratio_SPI
Ratio_DOM
MUS vs SPR
Ratio_SPR
Ratio_MUS
MUS vs SPI
Ratio_SPI
Ratio_MUS
SPR vs SPI
Ratio_SPI
Ratio_SPR

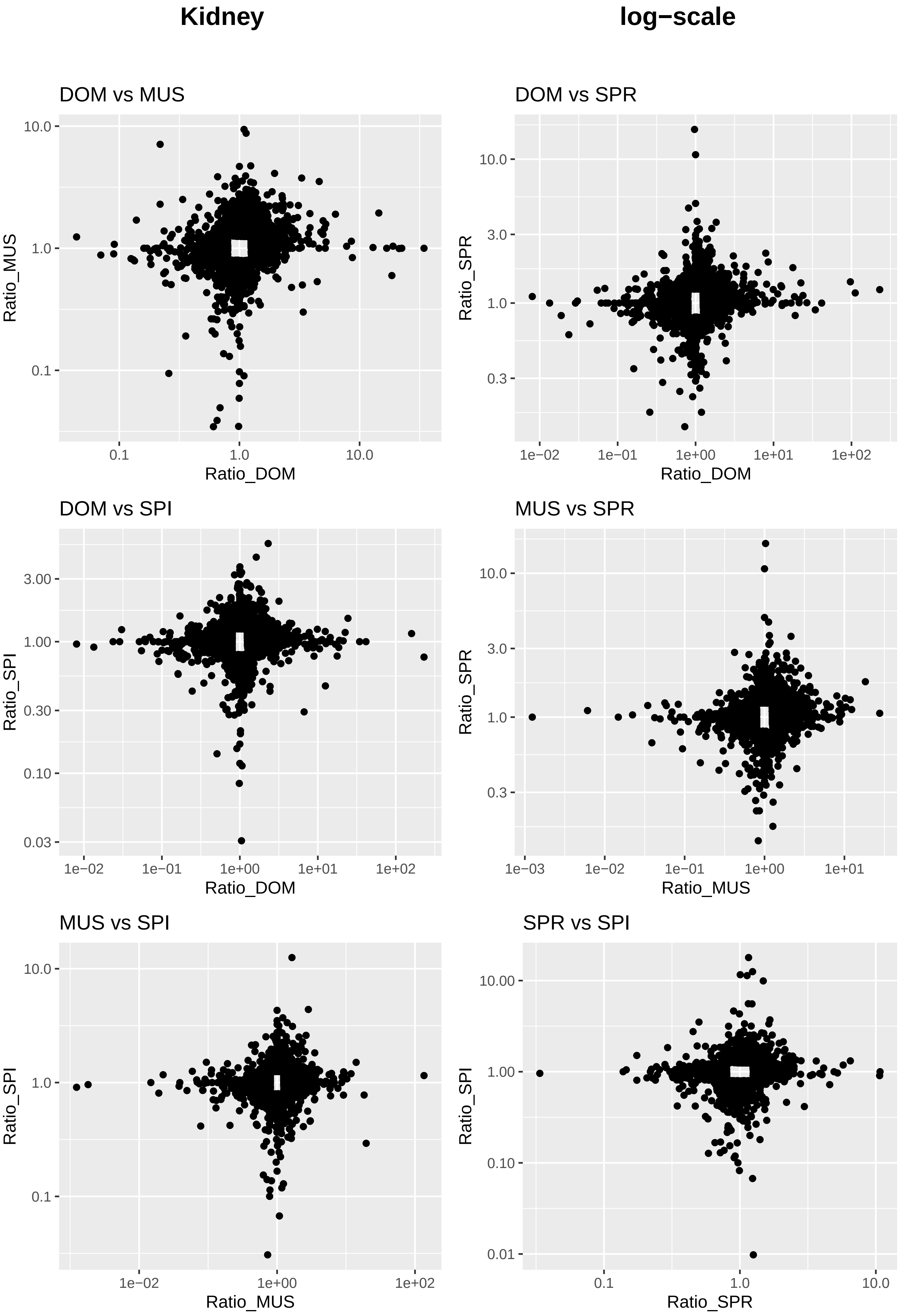
Kidney
log−scale
DOM vs MUS
Ratio_MUS
Ratio_DOM
10.0
1.0
0.1
DOM vs SPR
Ratio_SPR
Ratio_DOM
10.0
3.0
1.0
0.3
1e−02
1e−01
1e+00
1e+01
1e+02
DOM vs SPI
Ratio_SPI
Ratio_DOM
3.00
1.00
0.30
0.10
0.03
MUS vs SPR
Ratio_SPR
Ratio_MUS
1e−03
MUS vs SPI
Ratio_SPI
Ratio_MUS
SPR vs SPI
Ratio_SPI
Ratio_SPR
10.00
0.01

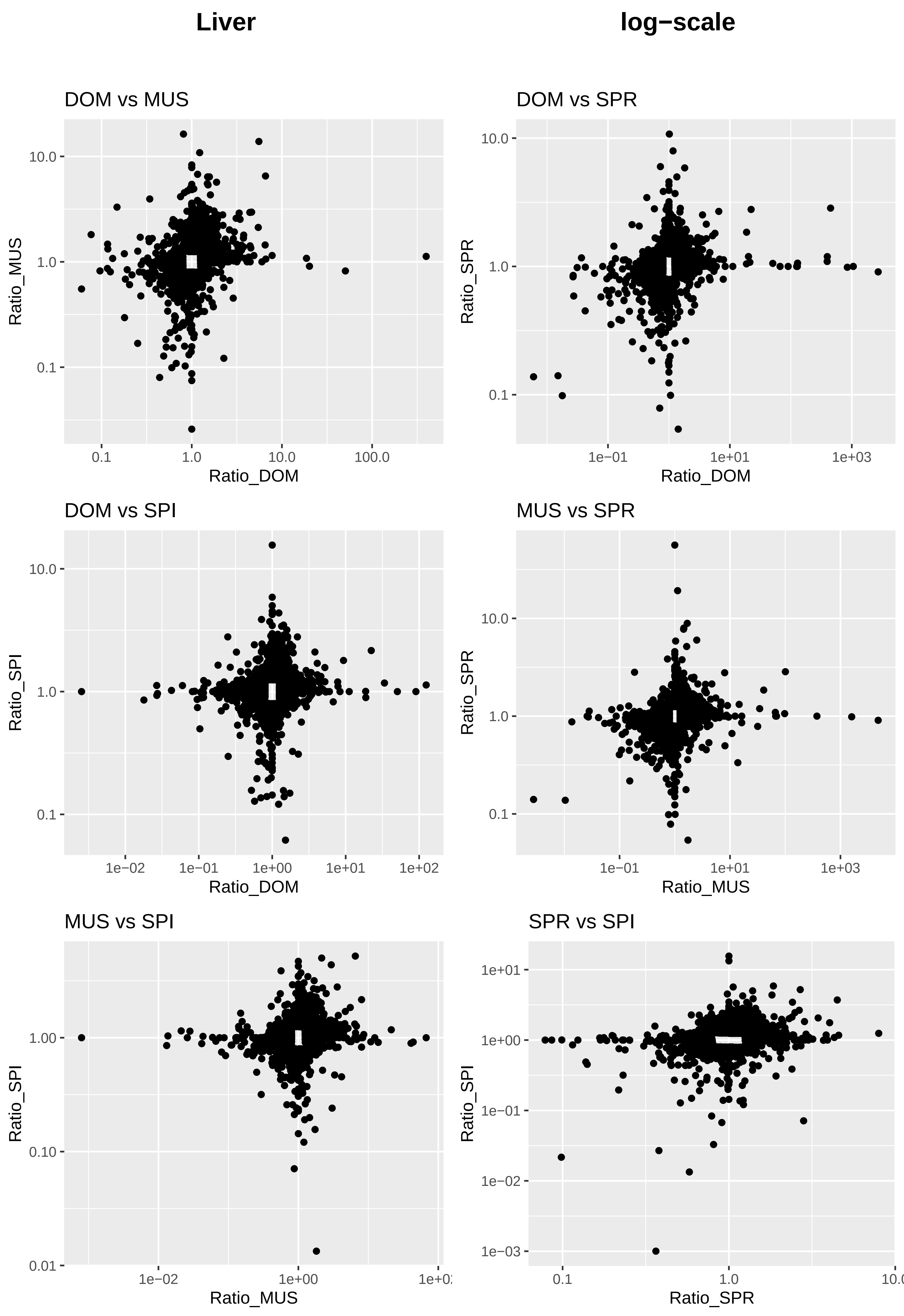
Liver
log−scale
DOM vs MUS
Ratio_MUS
Ratio_DOM
DOM vs SPR
Ratio_SPR
Ratio_DOM
DOM vs SPI
Ratio_SPI
Ratio_DOM
MUS vs SPR
Ratio_SPR
Ratio_MUS
MUS vs SPI
Ratio_SPI
Ratio_MUS
SPR vs SPI
Ratio_SPI
Ratio_SPR

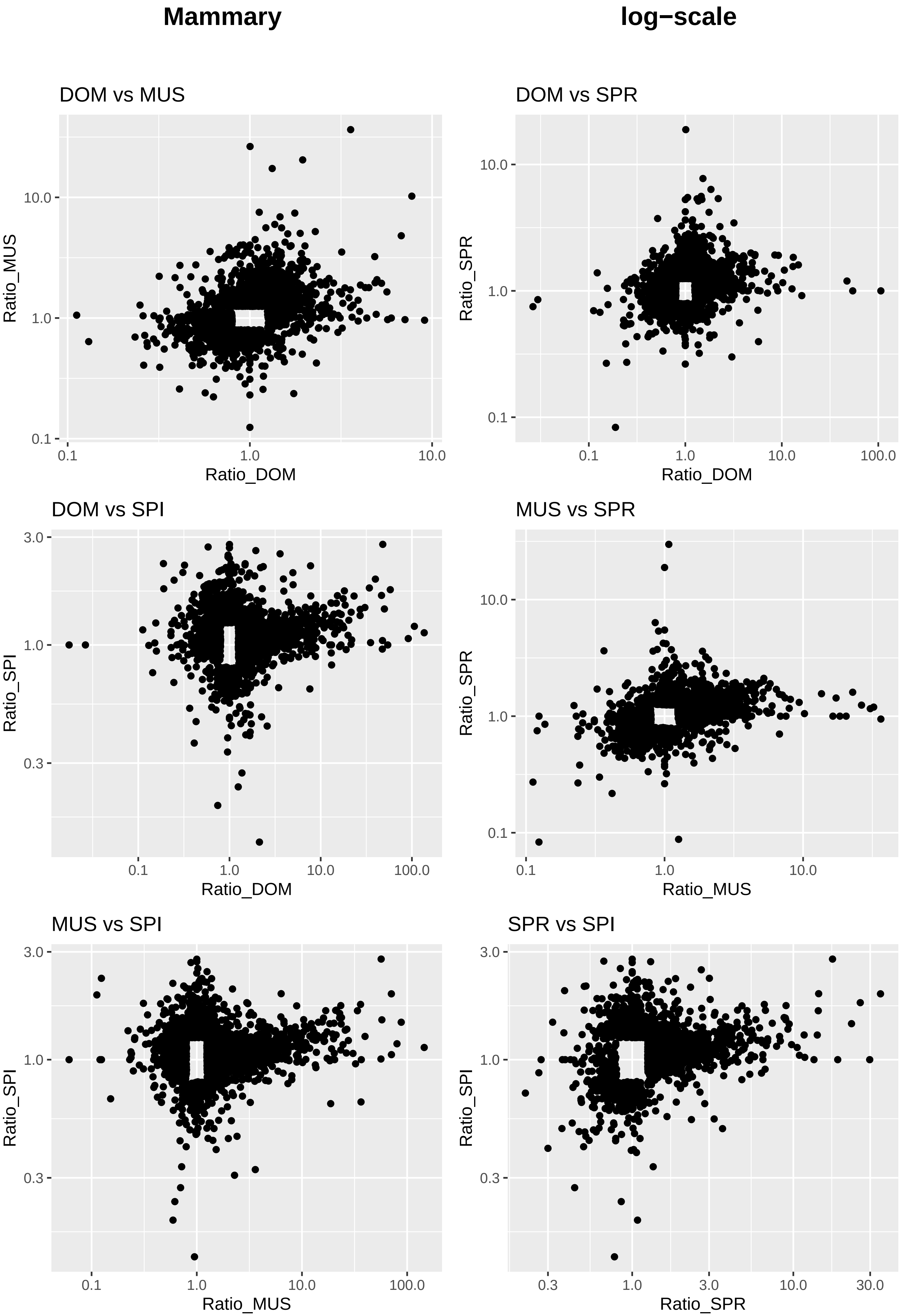
Mammary
log−scale
DOM vs MUS
Ratio_MUS
Ratio_DOM
DOM vs SPR
Ratio_SPR
Ratio_DOM
DOM vs SPI
Ratio_SPI
Ratio_DOM
MUS vs SPR
Ratio_SPR
Ratio_MUS
MUS vs SPI
Ratio_SPI
Ratio_MUS
SPR vs SPI
Ratio_SPI
Ratio_SPR

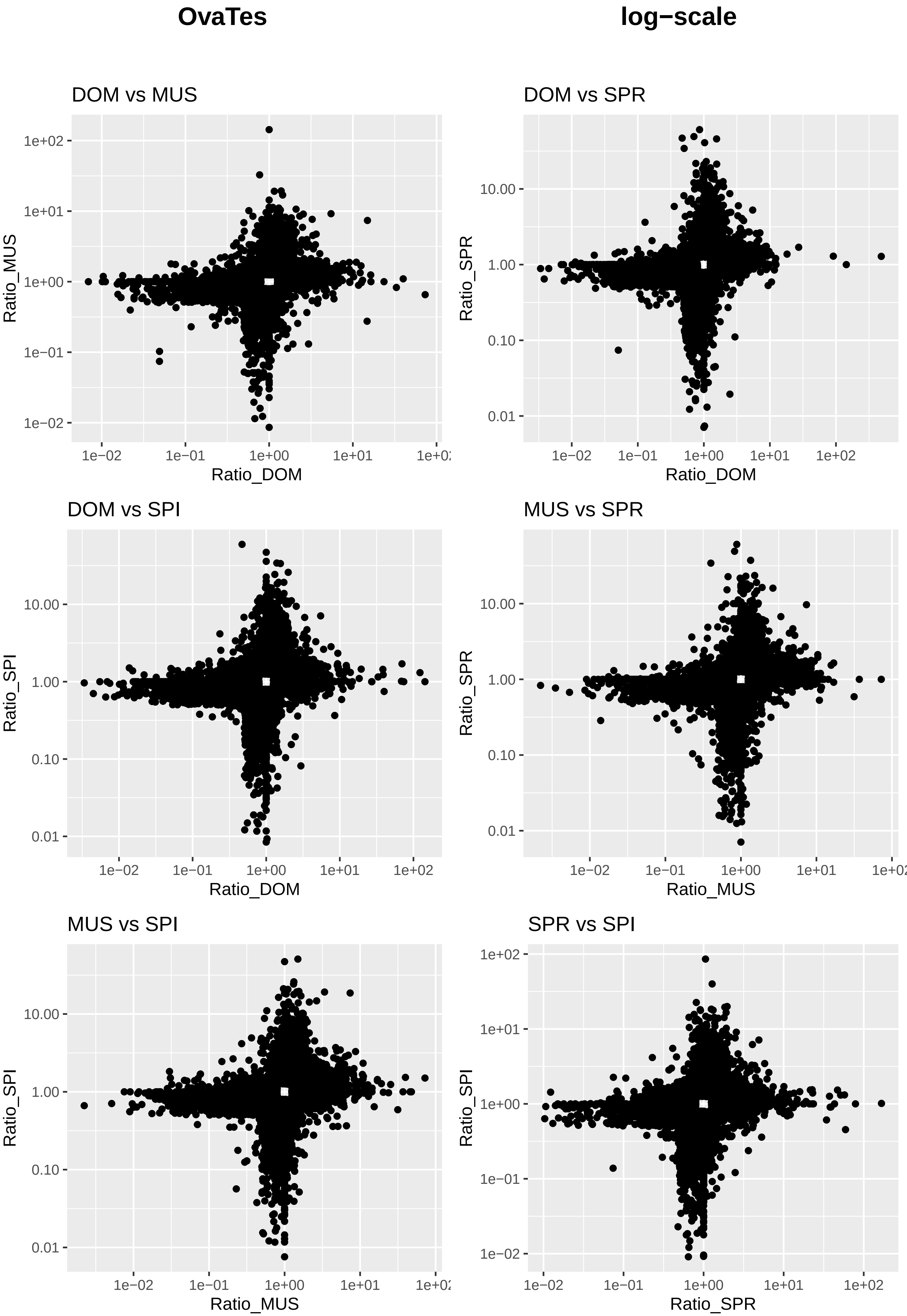

OvaTes
log−scale
DOM vs MUS
Ratio_MUS
Ratio_DOM
DOM vs SPR
Ratio_SPR
Ratio_DOM
DOM vs SPI
Ratio_SPI
Ratio_DOM
MUS vs SPR
Ratio_SPR
Ratio_MUS
MUS vs SPI
Ratio_SPI
Ratio_MUS
SPR vs SPI
Ratio_SPI
Ratio_SPR

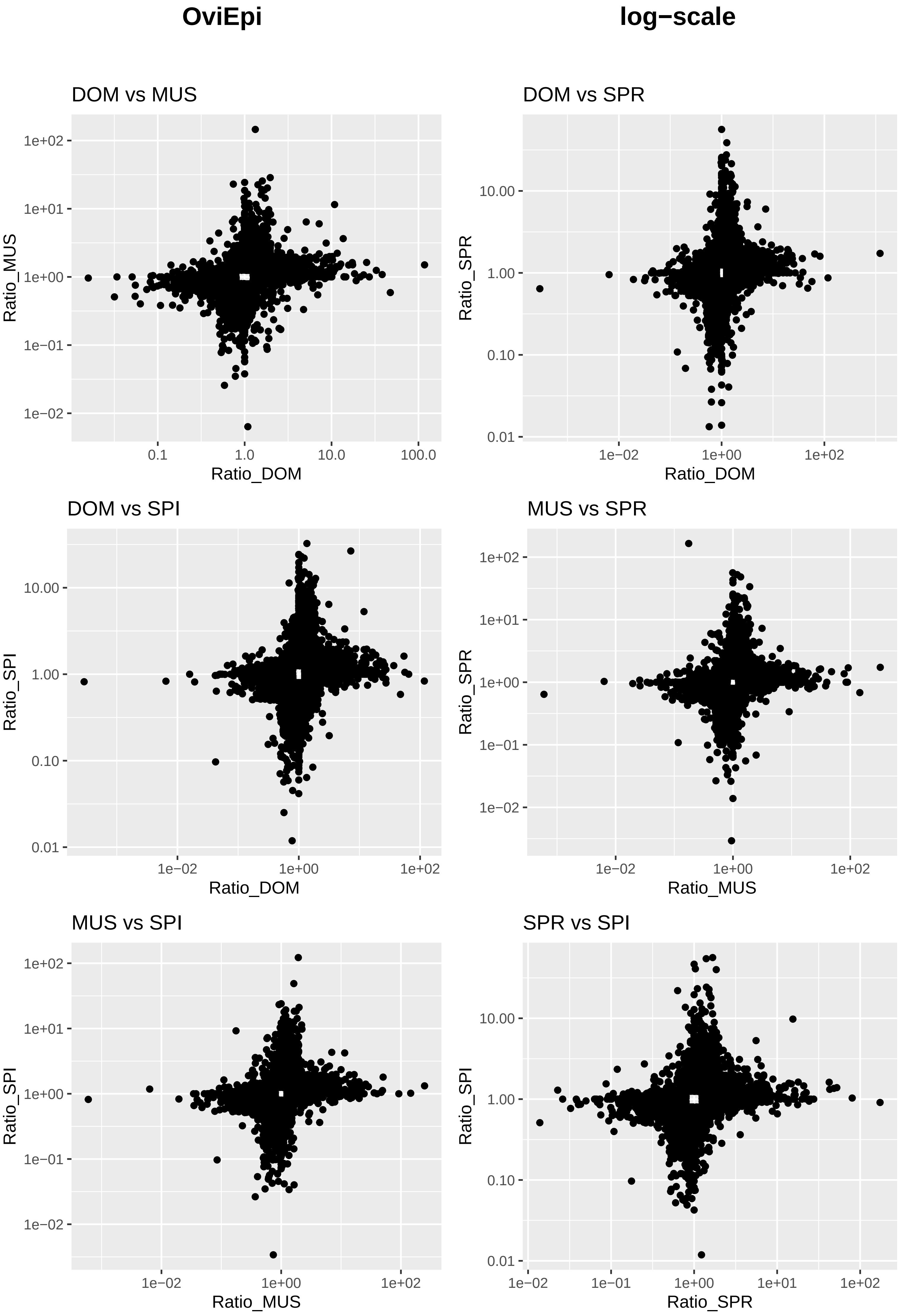

OviEpi
log−scale
DOM vs MUS
Ratio_MUS
1e+02
1e+01
1e+00
1e−01
1e−02
0.1
1.0
10.0
100.0
Ratio_DOM
DOM vs SPR
Ratio_SPR
10.00
1.00
0.10
0.01
1e−02
1e+00
1e+02
Ratio_DOM
DOM vs SPI
Ratio_SPI
10.00
1.00
0.10
0.01
1e−02
1e+00
1e+02
Ratio_DOM
MUS vs SPR
Ratio_SPR
1e+02
1e+01
1e+00
1e−01
1e−02
1e−02
1e+00
1e+02
Ratio_MUS
MUS vs SPI
Ratio_SPI
1e+02
1e+01
1e+00
1e−01
1e−02
1e−02
1e+00
1e+02
Ratio_MUS
SPR vs SPI
Ratio_SPI
10.00
1.00
0.10
0.01
1e−02
1e−01
1e+00
1e+01
1e+02
Ratio_SPR

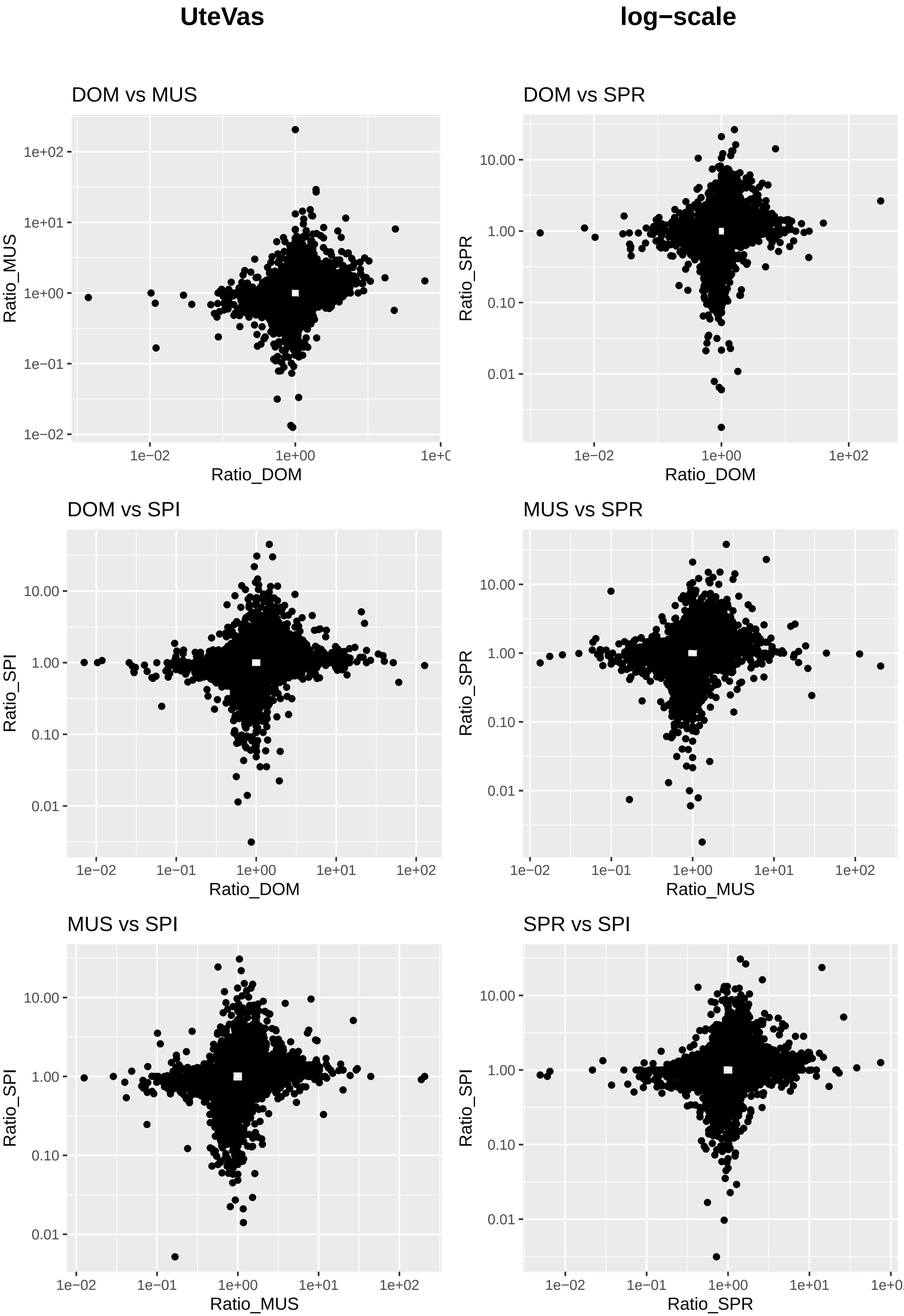

UteVas
log−scale
DOM vs MUS
DOM vs SPR
DOM vs SPI
MUS vs SPR
MUS vs SPI
SPR vs SPI
Ratio_DOM
Ratio_MUS
Ratio_SPR
Ratio_SPI
