# Supplementary material for: Fast evolutionary turnover and overlapping variances of sex-biased gene expression patterns defy a simple binary sex classification of somatic tissues
Source: eLife. 2025 Sep 17;13:RP99602. doi: 10.7554/eLife.99602 (PMC12443475; doi:10.7554/eLife.99602)
Supplement: Figure 4—source data 2. [file elife-99602-fig4-data2.pdf]

Figure 4 - figure supplement 1. Mean connectivity plots for the different organs for determining the soft threshold parameter β for the WGCNA module assignments.

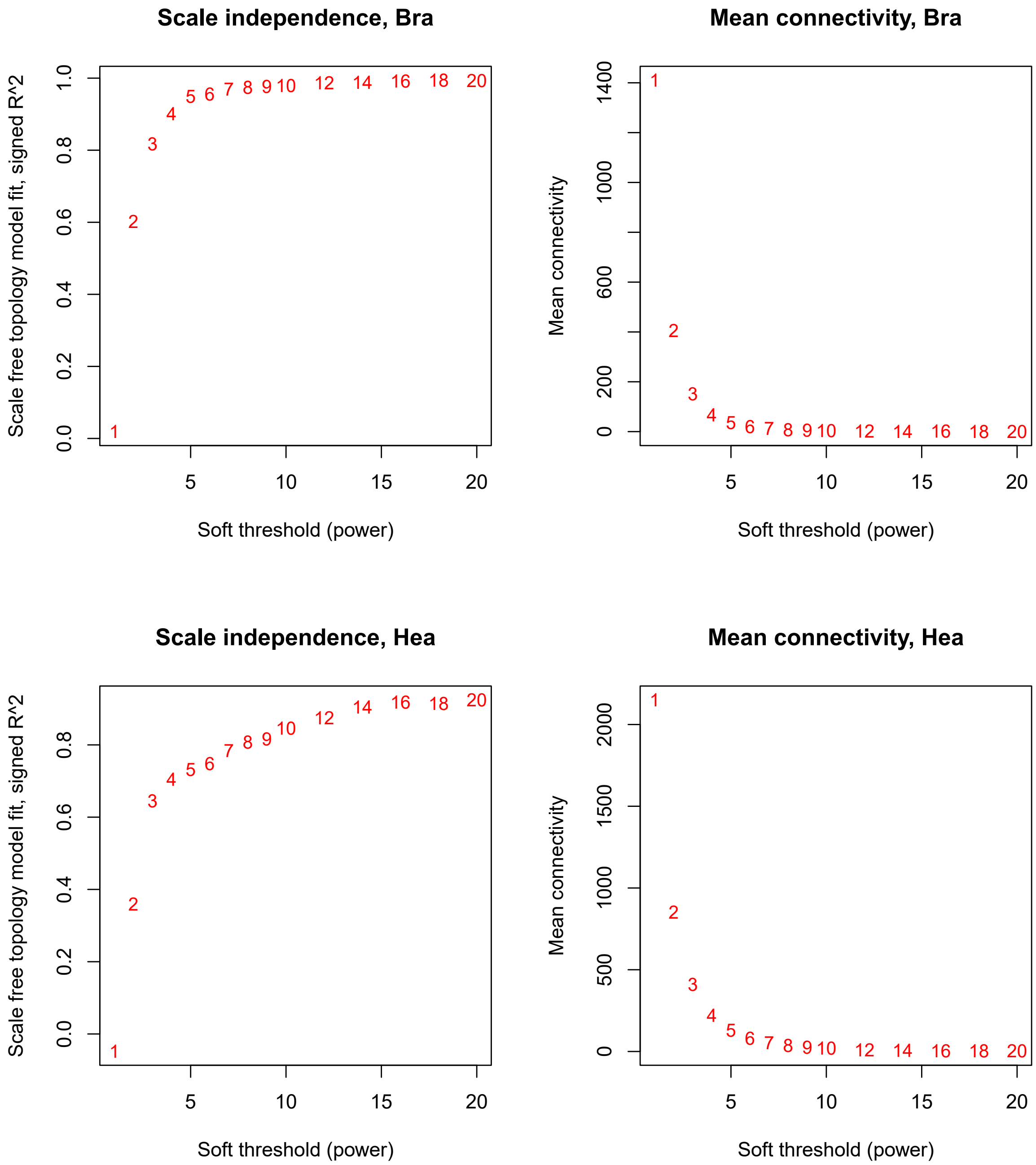

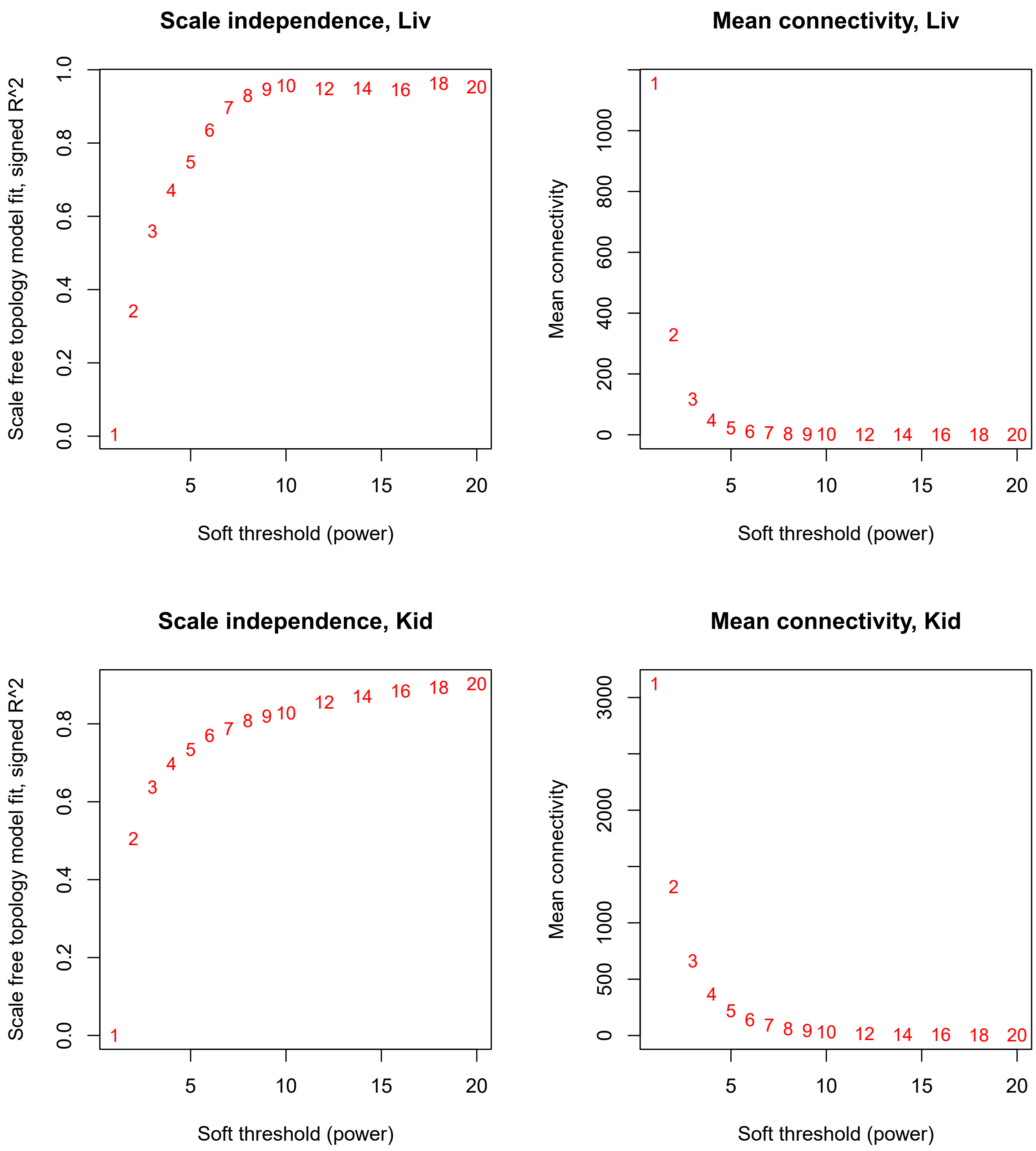
Scale independence, Liv
Mean connectivity, Liv
Scale independence, Kid
Mean connectivity, Kid
Scale free topology model fit, signed R^2
Mean connectivity
Soft threshold (power)
0.0
0.2
0.4
0.6
0.8
1.0
0
200
400
600
800
1000
500
2000
3000
5
10
15
20
1
2
3
4
5
6
7
8
9
10
12
14
16
18
20

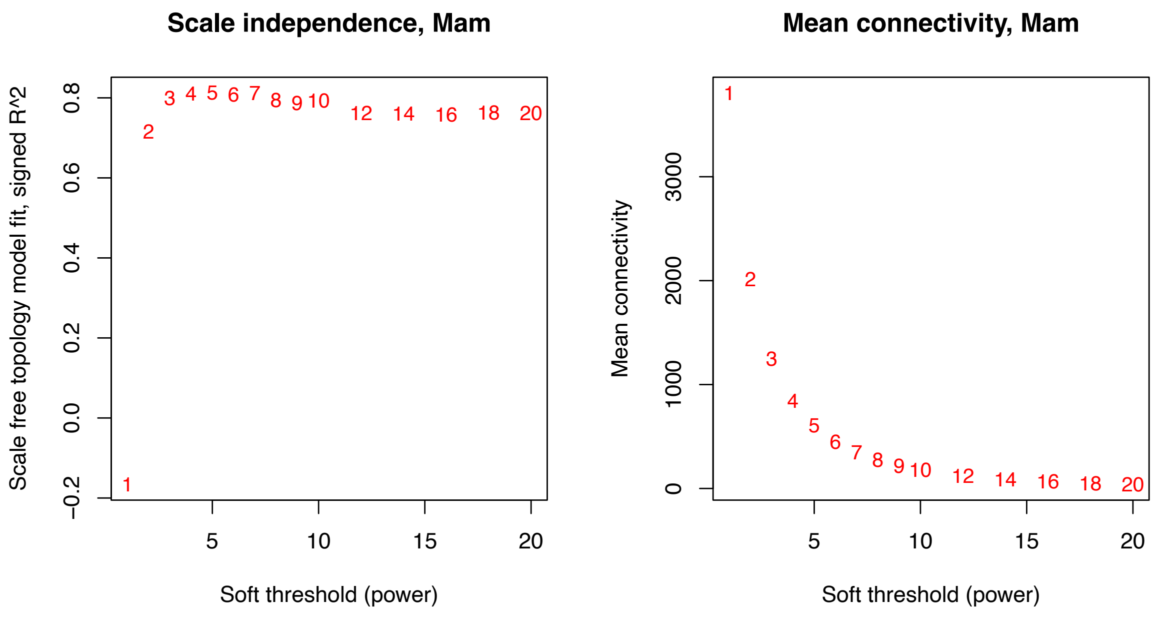
Scale independence, Mam
Scale free topology model fit, signed R^2
−0.2
0.0
0.2
0.4
0.6
0.8
1
2
3
4
5
6
7
8
9
10
12
14
16
18
20
5
10
15
20
Soft threshold (power)
Mean connectivity, Mam
Mean connectivity
0
1000
2000
3000
1
2
3
4
5
6
7
8
9
10
12
14
16
18
20
5
10
15
20
Soft threshold (power)
